# A novel use of small ballons to reduce the risk of subintimal hematoma formation during recanalization of chronic total occlusion: two case reports

**DOI:** 10.1186/s12872-022-02516-w

**Published:** 2022-02-26

**Authors:** Yanzhuo Ma, Xinxing Song, Lingfeng Kong, Gang Wang, Xiaoye Wang, Leisheng Ru

**Affiliations:** Department of Cardiology, Bethune International Peace Hospital, Shijiazhuang, Hebei China

**Keywords:** Antegrade approach, Chronic total occlusion, Percutaneous coronary intervention, Hematoma formation, Balloon occlusion

## Abstract

**Background:**

Subintimal hematoma remains a major challenge associated with unnecessary technical complexity, failure of the antegrade approach or imperfection of revascularization in percutaneous coronary intervention (PCI) for chronic total occlusion (CTO). Some techniques and devices release the hematoma after its formation. Here, we describe a novel use of small ballons to prevent the hematoma formation during antegrade approach in two cases.

**Case presentation:**

We report two cases of CTO-PCI in which balloon occlusion was successfully applied to prevent haematoma formation. The first case, a 72-year-old female with diabetes, was hospitalized because of unstable angina. Angiography showed right coronary artery (RCA) CTO, which initiated from the proximal part to the trifurcation at the distal part of the RCA. Considering the high likelihood and serious consequences of subintimal haematoma, a small balloon is employed to prevent subintimal hematoma formation. A balloon and microcatheter or double-lumen microcatheter are placed in the proximal coronary CTO lesion; then the balloon was dilated beside the catheter, most of the antegrade blood flow was sealed which reduced the likelihood of haematoma formation. The procedure was successfully completed without subintimal hematoma formation. The second case a 62-year-old male with unstable angina, was hospitalized for PCI. Angiography showed left anterior descending coronary artery CTO. Similar to case 1, we also used a small balloon to prevent hematoma formation. Both patients underwent PCI, which employed a small balloon to prevent hematoma formation and both procedures were successful without complications.

**Conclusion:**

In patients who underwent CTO-PCI, balloon occlusion offers an alternative for reducing the incidence of subintimal haematomas.

## Background

The antegrade approach plays an important role in percutaneous coronary intervention (PCI) for chronic total occlusion (CTO) [1, 2], which employs antegrade wire escalation (AWE) and antegrade dissection/re-entry (ADR) techniques to improve procedure success [3, 4]. Regarding the crossing strategies, it can be divided into intraplaque and subintimal tracking according to the guidewire position during CTO PCI [5, 6]. Subintimal tracking, particularly ADR (wire-based or device-based), often poses the higher risk of vessel dissection, whereas intraplaque tracking could also result in vessel dissection as the dedicated CTO guidewires are extra-stiff and are designed to penetrate the CTO plaque forcefully, so controlling the wire position precisely may be difficult, and after that, it could cause complications, such as intimal disruption or dissection [5, 7]. Vessel dissection predisposes to subintimal hematoma which can lead to uncontrolled vessel dissection, distal true lumen compression and wire re-entry impediment [8]; these factors may make the operator abort the antegrade approach and subsequently switch to other strategies.

**Fig. 1:**
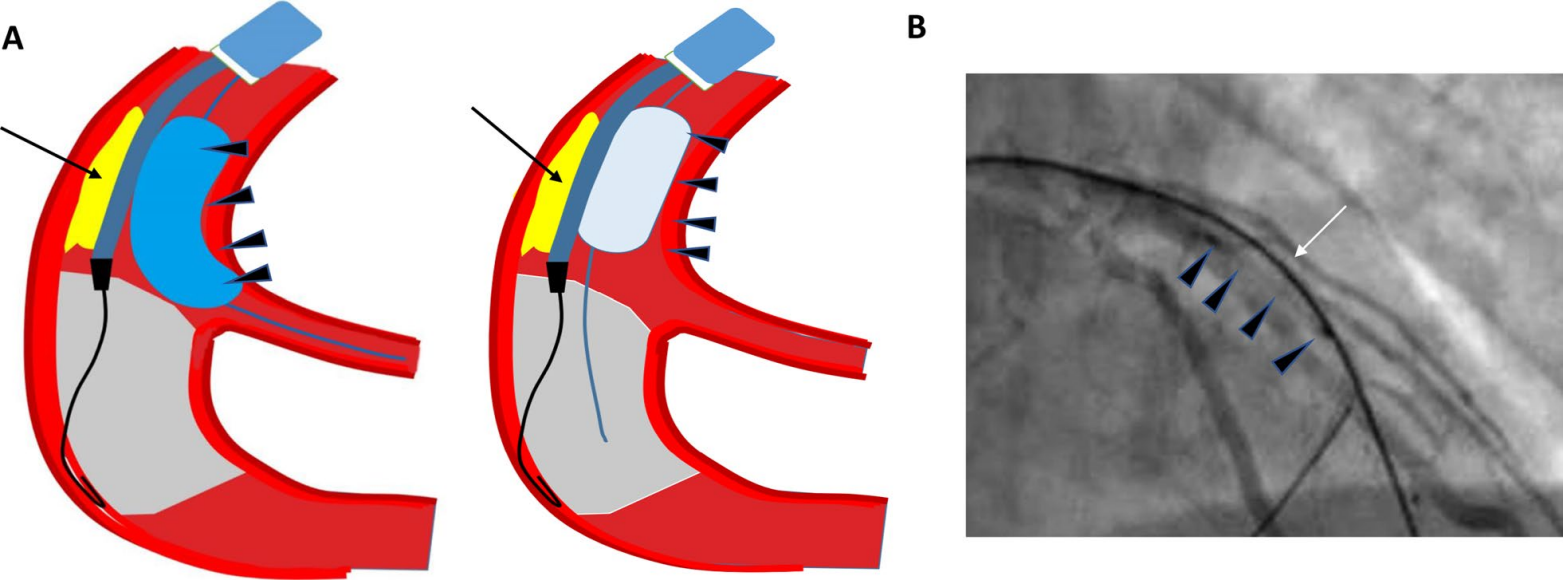
**A** Diagram of the novel technique, the balloon could be placed in the proximal of side branch for protection or in the main branch, **B** angiographic image of the novel technique. Black arrow: microcatheter; Triangle: dilated balloon

Some techniques and devices are used to alleviate subintimal hematoma; however, nowadays, there are few reports about preventing subintimal hematoma before its formation. As control of the antegrade blood flow into the subintimal space and the prevention of subintimal hematoma often indicate an easy puncture, crossing and re-entry for guidewire wiring, we describe a novel technique that employs a small balloon for dilating in the proximal CTO lesion using a microcatheter or a double-lumen microcatheter, so the antegrade blood flow is almost completely blocked and the puncture wire could be advanced via the microcatheter or double-lumen microcatheter to cross the occluded vessel simultaneously (Fig. 1A,B). Two case reports are presented to illustrate the efficiency and safety of this technique in an antegrade procedure for CTO PCI. The study was approved by the Research Ethics Committee of the institution in compliance with the Declaration of Helsinki, and that the participants provided written informed consent.

## Case presetation

### Case 1

**Fig. 2:**
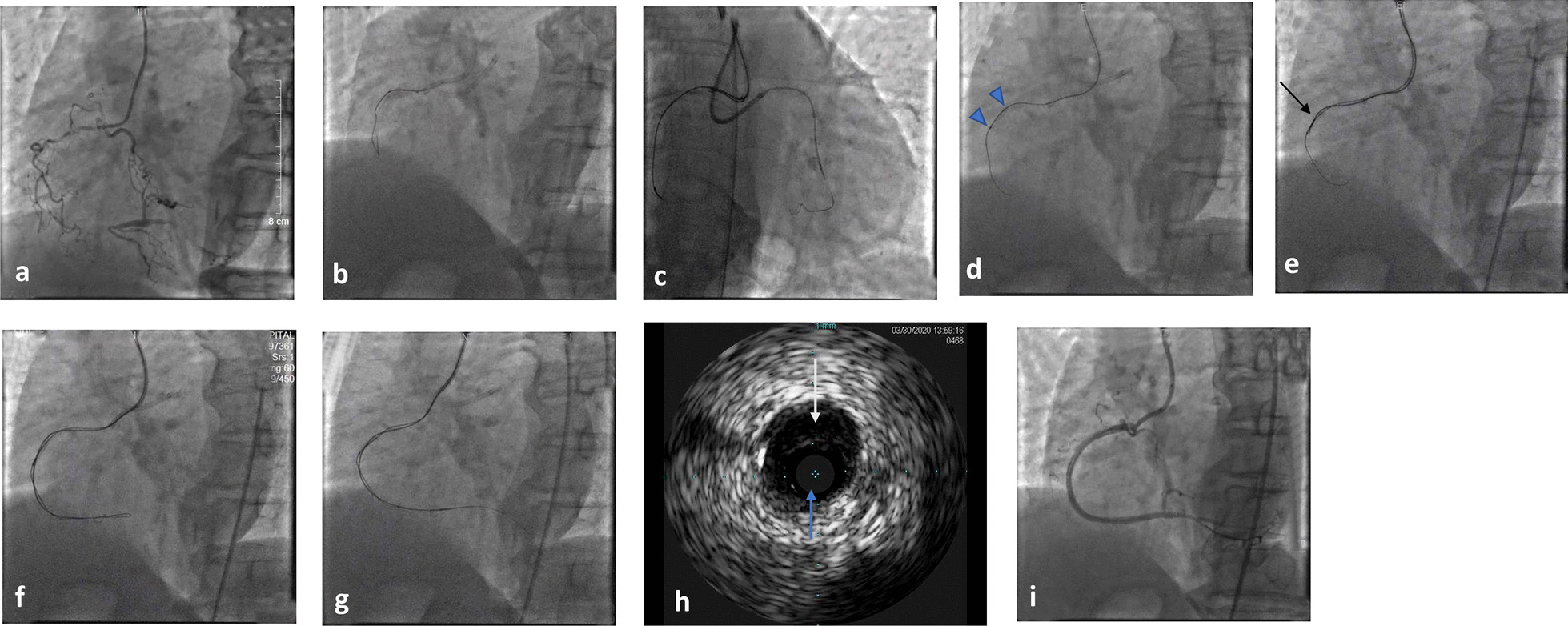
Angiographic images. **a** CTO of the RCA from the proximal to the posterolateral (PL) branch and posterior descending (PD) branch ostia. **b** antegrade guidewire was unable to cross. **c** the Sion black was advanced via Corsair 135 to cross the septal, but still failed. **d** a 2.0 × 15-mm balloon was advanced via the previous guidewire. **e** Corsair135 was introduced in parallel with the dilated balloon. **f** pilot200 was knuckled towards the PD branch. **g** the knuckled guidewire was advanced into the PD branch via Corsair 135. **h** part of the guidewire was in the subintimal area evidenced by IVUS. **i** stents were implanted and the final angiogram was excellent. Black arrow: microcatheter; White arrow: true lumen; Blue arrow: false lumen; Triangle: dilated balloon

A 72-year-old female with unstable angina had a history of hypertension dyslipidemia and diabetes. Dual angiography showed a right coronary artery(RCA-) CTO, which initiated from the proximal part to the trifurcation at the distal part of the RCA (Fig. 2a). A 6-F EBU3.5 guiding catheter (Medtronic Inc., Minneapolis, MN, USA) was placed at the left coronary artery (LCA) through the radial artery, and a 7F AL1 guiding catheter (Cordis, Santa Clara, CA, USA) was settled at the RCA via the femoral artery. AWE was proceeded first but failed (Fig. 2b). Then, a retrograde intervention was performed; however, the procedure also failed to cross the collateral connections (Fig. 2c). Finally, we switched to the ADR technique. A hematoma is one of the most important factors in the success of the ADR; the less hematoma formation there is, the more successfull of the ADR is, thus, we placed a 2.0 × 15 mm compliant balloon (Boston Scientific, Marlborough, MA, USA) at the proximal part of the CTO via the previous guidewire (Fig. 2d) and paralleled it with a microcatheter (Corsair135cm). The balloon inflated at 10 atm and the Corsair135 at the proximal CTO lesion almost completely sealed the blood inflow (Fig. 2e). A pilot200 through the corsair135 penetrated the initial CTO body and then was knuckled to the distal part of the CTO body (Fig. 2f). No subintimal hematoma was observed, evidenced by angiography, and an escalated CTO wire CP8-20 penetrated to the distal true lumen (Fig. 2g). The total wiring time for the RCA was 47 min, and part of the guidewire was passed through the subintimal space to the distal of RAC evidenced by intravascular ultrasound (IVUS) (Fig. 2h). Four stents were implanted, and the final angiogram was excellent (Fig. 2i).

### Case 2

**Fig. 3:**
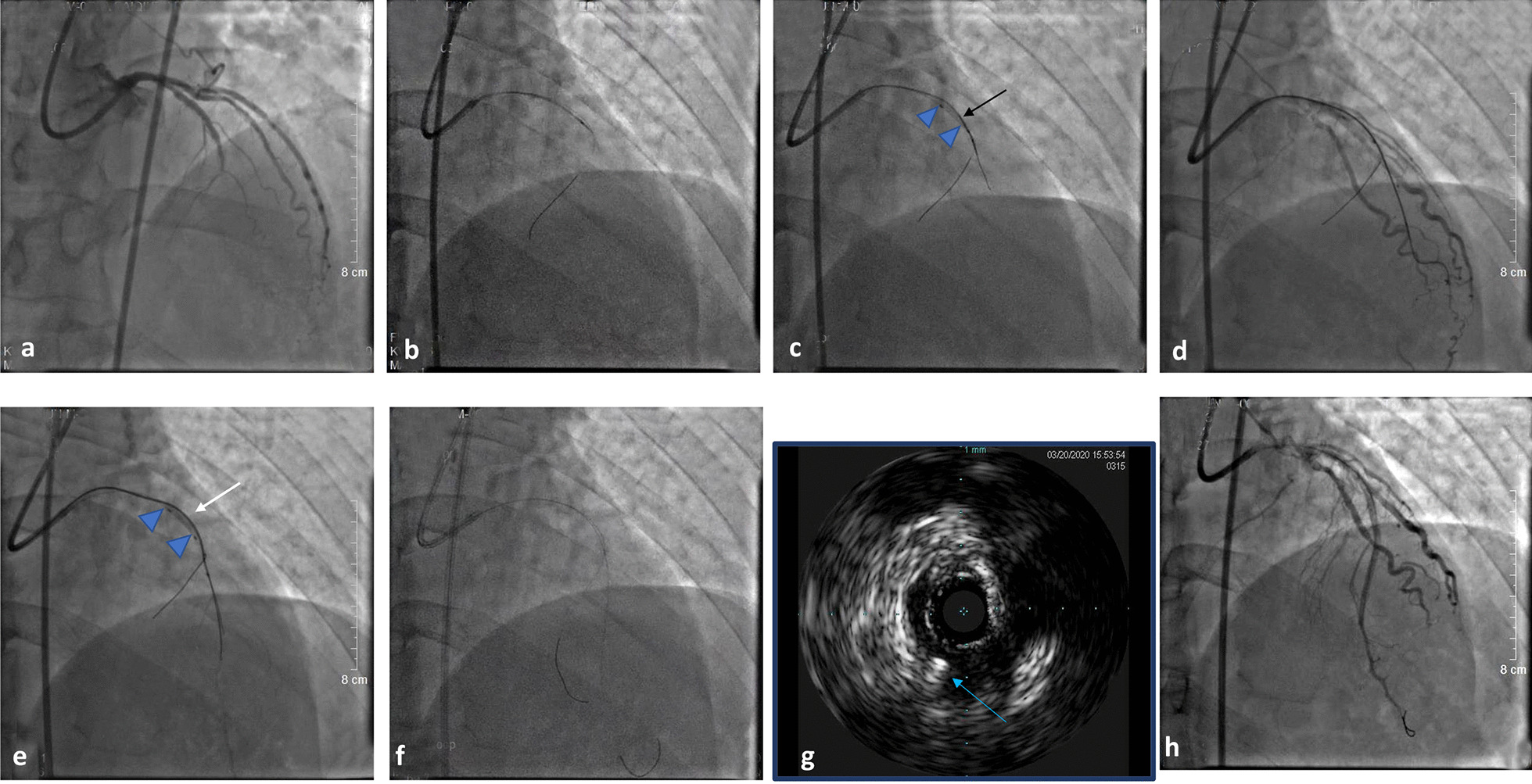
Angiographic images. **a** CTO of the LAD from the middle to the distal. **b** a balloon and a corsair135 were both introduced to the proximal LAD. **c** we performed balloon dilation with a 2.0 × 15-mm balloon. **d** the guidewire was advanced into the subintimal place. **e** a Crusade was used to facilitate the Gaia III into the distal true lumen by using the parallel wire technique. **f** the Gaia III passed to the distal LAD. **g** The final angiographic result was excellent. Black arrow: microcatheter; White arrow: double-lumen microcatheter; Blue arrow: Pilot 200; Triangle: dilated balloon

A 62-year-old male with unstable angina had a history of hypertension. Anterograde angiography showed a left anterior descending artery(LAD) CTO with a well-developed ipsilateral collateral circulation, which initiated from the medial partto the distal part of the LAD (Fig. 3a). A 6F EBU 3.5 guiding catheter was placed at the ostium of the LCA. A Runthrough NS achieved to the septal vessel introduced a 2.0 × 15 mm compliant balloon (Boston Scientific, Marlborough, MA, USA) to the proximal LAD, while another Runthrough NS introduced a microcatheter, Corsair135 to the initial of the CTO lesion (Fig. 3b). The 2.0 × 15 mm balloon was dilated with 10 atm at the proximal part of the LAD to block antegrade blood flow (Fig. 3c). Meanwhile, CTO wires, such as XT-A and pilot 200, were introduced through the microcatheter to penetrate the CTO body; unsurprisingly, parts of the wire jumped into the subintimal space (Fig. 3d). Here and now, the parallel wiring technique might be useful. With the assistance afforded by the crusade, a double-lumen microcatheter, Gaia III was advanced step by step into the distal true lumen, which was verified by contrast angiography (Fig. 3e), and then the Gaia III was passed to the distal LAD (Fig, 3f). The total wiring time for the RCA was 35 min. Almost no hematoma formation was observed. IVUS was introduced to the LAD and showed that parts of the pilot 200 were in the subintimal space (Fig. 3g). The final angiographic image showed an excellent result after two stents were deployed (Fig. 3h).

## Discussion and conclusions

Remarkable improvements have been made in CTO-PCI with the development of wiring techniques, equipment and operator experience over the last decades [1, 9]. The antegrade approach is one of the favoured strategies of the hybrid algorithm for CTO revascularization and is frequently associated with subintimal tracking. However, guidewire entry into the subintimal space increases the risk of vessel dissection that could lead to the formation of subintimal hematomas. Besides, intraplaque tracking could also cause intimal injury, even vessel dissection or hematoma, although the rate of vessel dissection may be lower than that when compared with subintimal tracking is employed.

Keeping the dissection and subsequent hematomas as short as possible when they occur is desirable [10–12]. However, traditional treatment, including balloon angioplasty and/or stenting would result in longer vessel dissection planes, more side branch occlusion and less likelihood of recanalization success, which influence the procedural and follow-up clinical outcomes of CTO PCI by affecting the distal outflow and blood flow recovery [13]. Several techniques have been developed to decompress subintimal hematoma, however, they require extra devices [14–17]. Therefore, the blockage of antegrade blood flow into the subintimal space is a key step in minimizing the risk of hematoma formation, thus enhancing the chance of procedure success. Some devices have been used to reduce the incidence of hematoma formation. Using Guide extension catheters that are the same size as the guiding catheter could reduce the amount of blood inflow, however, a small amount of blood flow into the CTO vessel could be reduced due to the space between the extension catheter and the guiding catheter. Furthermore, guide extension catheters are specially needed, which could increase the procedure time and cost. Some operators recommend routinely using haematoma decompressing techniques, including the stick and swap and subintimal transcatheter withdrawal (STRAW) techniques using a Stingray balloon [18], such as keeping one or two 2.5–3.5 mm balloons besides the Stingray LP in an 8-F guiding catheter to occlude the proximal CTO vessel; nevertheless, these techniques require special devices and/or require larger guiding catheters.

In the two cases, we described a novel use of small balloons to reduce the risk of hematoma formation, which is performed at the beginning of the antegrade procedure and lasts until the guidewire is advanced into the distal true lumen of the occluded vessel. This technique provided a better control of the antegrade blood flow and prevented the formation of subintimal hematomas without requiring special devices. An appropriate balloon chosen according to the characteristics of the target vessel and the lesion was advanced along a microcatheter or double-lumen microcatheter in the proximal CTO vessel, and then, balloon dilation was performed, so the antegrade blood flow was almost completely sealed after balloon dilation as the space between the dilated balloon and catheter barely existed. The puncture guidewire could be manipulated smoothly though the microcatheter or double-lumen microcatheter without being affected by the small dilated balloon. In addition, the dilated balloon also provided extra anchor support for antegrade wire crossing. Moreover, the dilated small balloon using adjustable inflation pressure had little effect on the advancement of the catheter. Most importantly, no complications and no evidence of subintimal hematoma were observed.

Apart from ADR intervention, which conventionally uses the subintimal space for wire re-entry, part of the puncture guidewire may cross through the subintimal place even when we intended to perform intraplaque tracking, similar to that in case 2. Hence, using this novel technique for favourably blocking the antegrade blood flow during antegrade intervention, regardless of intraplaque tracking or subintimal tracking, can help reduce the risk of subintimal hematoma formation. This allows the operators to perform the antegrade intervention more effectively and ambitious without considering the adverse effects caused by hematoma.

However, this technique has some limitations. It cannot be used in ostium CTO vessel initially; since the dilated balloon and catheter are unlikely to be well anchored at this position.

In conclusion, this technique provides a safe and effective solution for preventing hematomas encountered during antegrade intervention. It is cost-effective and easy to perform. We suggest routine use of this technique during antegrade approach given the wide availability of a balloon.

## Data Availability

Data associated with this manuscript are not publicly available, but can be made available by the corresponding author upon reasonable request.

## Abbreviations

PCI: Percutaneous coronary intervention
CTO: Chronic total occlusion
RCA: Right coronary artery
AWE: Antegrade wire escalation
ADR: Antegrade dissection/re-entry
STRAW: Subintimal transcatheter withdrawal
